# Death of Mr. Lynn

**Published:** 1837-10-01

**Authors:** 


### Obituary.

### 1. Death of Mr. Lynn.

Mr. Lynn, who was long surgeon to the Westminster Hospital, and was one of the few remaining samples of the 'old school', died in June, of the present year, in the 84th year of his age.

